# Global Meta-Analysis of Transcriptomics Studies

**DOI:** 10.1371/journal.pone.0089318

**Published:** 2014-02-26

**Authors:** José Caldas, Susana Vinga

**Affiliations:** 1 INESC-ID, Instituto de Engenharia de Sistemas e Computadores, Investigação e Desenvolvimento, Lisboa, Portugal; 2 IDMEC/LAETA, Instituto Superior Técnico, Universidade de Lisboa, Lisboa, Portugal; Medical University Hamburg, University Heart Center, Germany

## Abstract

Transcriptomics meta-analysis aims at re-using existing data to derive novel biological hypotheses, and is motivated by the public availability of a large number of independent studies. Current methods are based on breaking down studies into multiple comparisons between phenotypes (e.g. disease vs. healthy), based on the studies' experimental designs, followed by computing the overlap between the resulting differential expression signatures. While useful, in this methodology each study yields multiple independent phenotype comparisons, and connections are established not between studies, but rather between subsets of the studies corresponding to phenotype comparisons. We propose a rank-based statistical meta-analysis framework that establishes global connections between transcriptomics studies without breaking down studies into sets of phenotype comparisons. By using a rank product method, our framework extracts global features from each study, corresponding to genes that are consistently among the most expressed or differentially expressed genes in that study. Those features are then statistically modelled via a term-frequency inverse-document frequency (TF-IDF) model, which is then used for connecting studies. Our framework is fast and parameter-free; when applied to large collections of *Homo sapiens* and *Streptococcus pneumoniae* transcriptomics studies, it performs better than similarity-based approaches in retrieving related studies, using a Medical Subject Headings gold standard. Finally, we highlight via case studies how the framework can be used to derive novel biological hypotheses regarding related studies and the genes that drive those connections. Our proposed statistical framework shows that it is possible to perform a meta-analysis of transcriptomics studies with arbitrary experimental designs by deriving global expression features rather than decomposing studies into multiple phenotype comparisons.

## Introduction

Meta-analysis consists of aggregating the outcome of multiple studies in order to derive robust and reproducible results [1]. In DNA microarray transcriptomics, the availability of large-scale public databases such as ArrayExpress has encouraged the development of meta-analysis methods [2]. The meta-analysis paradigm has been successfully used in a variety of applications, including oncogenomics [3], drug repurposing [4], disease diagnosis [5], and mapping of tissue and condition-specific expression signatures [6]. Additionally, web-based tools have been developed for facilitating the exploration of large collections of transcriptomics studies [7]–[9]. For recent reviews of the field, see *e.g.* [10] or [11].

**Figure 1:**
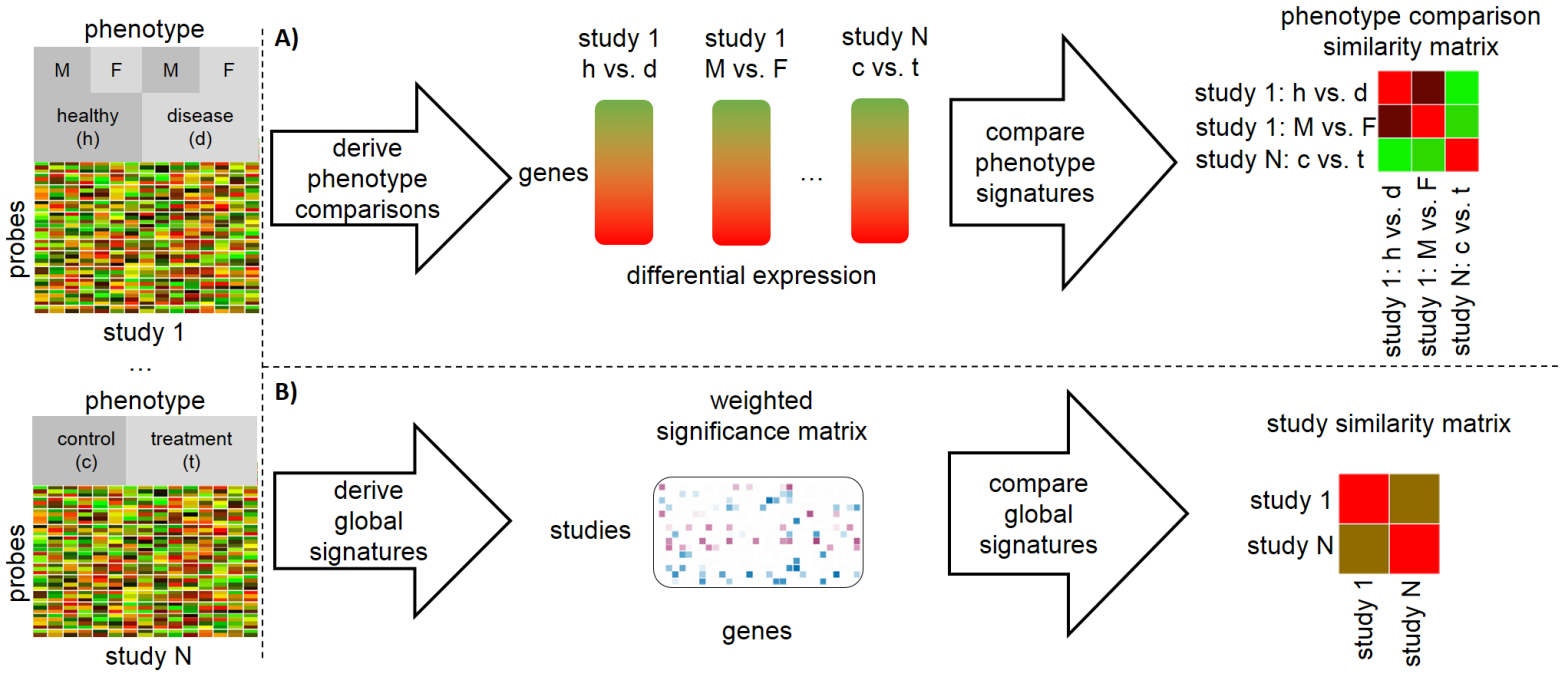
Summary of the steps performed (A) in the differential expression meta-analysis paradigm and (B) in our proposed framework. Two studies from a collection of 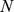 independent studies are shown. One of the studies includes experimental annotations about gender (Male or Female) and disease status (Healthy or Disease), while the other study includes experimental annotations indicating whether each sample comes from a Control or a Treatment. In the differential expression approach each study is broken down into a set of comparisons between phenotypes, with the first study yielding two comparisons and the second study yielding a single comparison; genes are ordered according to a differential expression measure, *e.g.*, log-ratios, which are used as a basis for computing the similarities between phenotype comparisons. In the proposed approach, one first decides if the analysis is based on absolute expression or differential expression. Then, in the case that one is interested in a differential expression analysis, the expression in every non-control sample (*e.g.* Treatment or Disease) is computed relative to the corresponding control sample. Afterwards, the rank product method is used to compute global, study-wide features corresponding to genes that possess a consistently high expression or differential expression in a given study. These global features are statistically modelled and used as a basis for deriving similarities between studies. The connections are therefore established not between subsets of studies, but rather directly between studies.

The challenges involved in performing meta-analysis stem from the intrinsic complexity of biological phenotypes, as well as from the observed heterogeneity between independent studies, namely the use of multiple microarray platforms, biological samples, and experimental procedures. In order to minimize the impact of those factors, meta-analysis methods typically focus on examining differential expression signatures across studies. Figure 1a illustrates this approach. This consists of first breaking down each study into a set of comparisons between phenotypes and then deriving a differential expression signature from each phenotype comparison. Connections between phenotype comparisons are then established based on the similarity between the corresponding differential expression signatures. Potential similarity measures include for instance correlation-based methods or probabilistic relevance measures [8],[9].

Meta-analysis approaches based on decomposing studies into comparisons between phenotypes, while successful, are critically hampered by the lack of extensive experimental and clinical annotations about the samples in each study. They also do not infer global connections between studies, rather detecting similarities between subsets of the studies corresponding to specific phenotype comparisons (*cf.* Fig.1a). While this latter aspect is not necessarily a disadvantage, an important methodological open question in transcriptomics meta-analysis is whether it is possible to aggregate and summarize all of the expression data within each study, regardless of the study's specific experimental design, with the aim of directly establishing global connections between independent studies. To the best of our knowledge, no general methods have been proposed with this purpose in mind.

While gene or pathway-oriented statistical tests [12] have been frequently used in the context of differential expression-based meta-analysis frameworks, they rely on decomposing each study into a set of comparisons between phenotypes. On the other hand, standard similarity measures such as Spearman correlation may be combined with linkage criteria to establish an overall similarity measure between studies. For instance, the global similarity between two studies may be computed as the mean Spearman correlation between microarrays from the two studies. However, this approach merely aggregates similarity measures between study subsets; our aim is rather to devise a method for globally encoding each study with features that represent the study's relevant expression signatures, allowing studies to be globally connected on the basis of those features. Additionally, the global features can be used to guide the interpretation of the inferred connections, which helps to formulate posterior biological hypotheses.

We propose a statistical meta-analysis framework for establishing global connections between transcriptomics studies, which does not require breaking down the studies into sets of phenotype comparisons. Intuitively, our approach detects the genes within each study that consistently possess high absolute or differential expression (for one-channel arrays) or expression ratio (for two-channel arrays) values across the arrays in that study. If the aim is to analyze absolute expression values in one-channel arrays, our approach does not make use of each study's experimental annotation. On the other hand, if the aim is to analyze differential expression values in one-channel arrays, our approach simply requires an indication of which samples in the study are used as a control. Connections between studies are then inferred and interpreted on the basis of shared relevant genes, via a vector space model. Figure 1b summarizes the flowchart of the proposed framework.

Our framework partly relies on using the non-parametric Rank Product method [13] to aggregate gene expression data from all samples in any given study. The rank product method has been previously shown to be a useful statistic for aggregating differential expression fold-change measures across independent control-vs-treatment studies [14]. In the present paper, we apply the rank product method in a vastly different manner. Instead of restricting ourselves to simple control-vs-treatment studies, we consider studies with arbitrary experimental designs. Also, we do not use the rank product method to directly aggregate data from independent studies; rather, we use the rank product method to obtain a measure of consistent expression for every gene *within* each study, thus deriving an encoding for every study which is then used as a basis for matching independent studies according to their similarity. In effect, in our setting it is not possible or meaningful to apply the rank product method as described by Hong and Breitling [14], because we do not have an *a priori* list of related studies with equivalent experimental designs; we instead have a large, heterogeneous collection of studies with varying experimental designs that we want to mine for interesting connections.

We apply our framework to large, heterogeneous collections of transcriptomics studies from human and *Streptococcus pneumoniae*. We perform an analysis both on absolute and differential expression data, and also provide a strategy for harmonizing data from multiple platforms and *S. pneumoniae* strains. We propose a quantitative validation strategy that assesses the quality of the inferred connections by verifying if the connected studies share Medical Subject Headings (MeSH) terms [15]. Using this strategy, we show that our method outperforms standard correlation-based measures in terms of connecting studies that are effectively related. Finally, we provide illustrative case studies of applying our method to both study collections.

## Results and Discussion

**Figure 2:**
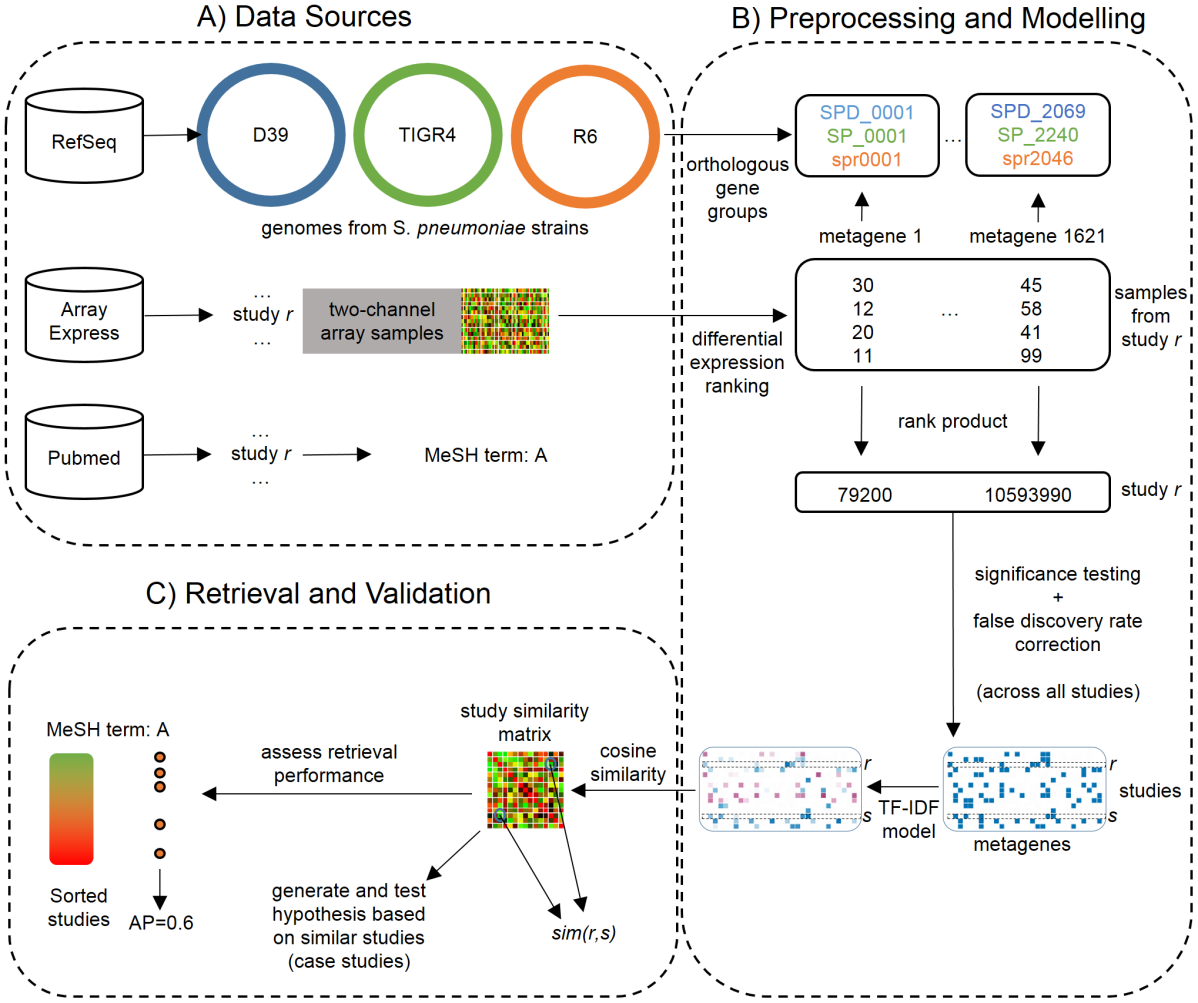
Detailed flowchart for the proposed framework. For succinctness, we illustrate the steps as applied to the *Streptococcus pneumoniae* two-channel array data collection. In stage (A), we download data from three souces: RefSeq, for bacterial genomes from multiple *S. pneumoniae* strains; ArrayExpress, for two-channel microarray transcriptomics studies along with the corresponding experimental annotations; PubMed, for the MeSH annotations associated with each study's corresponding publication. Stage (B) consists of multiple preprocessing and modeling steps. We start by mapping orthologous genes from different strains to the same so-called metagene. For each transcriptomics study, we rank metagenes in each sample according to their absolute log-ratio. Then, for each study and metagene we take the product of its ranks across the study's samples, derive a corresponding 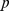-value, and binarize 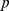-values accounting for multiple hypothesis testing. This yields a metagenes 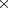 studies binary matrix that indicates for every metagene-study pair if the metagene is significant in the corresponding study. The last step in stage (B) is to convert the binary matrix into a weighted matrix via TF-IDF modeling. This consists simply of replacing ones in the binary matrix by a weight that is indicative of the corresponding metagene's information content. In stage (C), we apply the cosine similarity measure to connect studies on the basis of their TF-IDF vector-space encoding. We then assess the performance of the framework by quantifying the extent to which related studies are found to be similar, using MeSH terms as a gold standard. We also inspect the similarity matrix to detect interesting case studies that provide novel biological hypotheses.

In this section we provide an analysis of our proposed framework. Figure 2 displays a detailed methodology flowchart. We assess our framework in two ways: First, we measure the ability of the framework to detect connections between studies that are effectively related, using MeSH terms assigned to each study as a gold standard; second, we provide multiple case studies that show how the framework can be used as an exploratory tool to detect interesting connections between transcriptomics studies and generate novel biological hypotheses.

The results and accompanying R software are available from http://kdbio.inesc-id.pt/~svinga/meta/.

### Method Comparison

We applied the MeSH test setting outlined in the Methods section to compare our framework to alternative approaches in three contexts: one-channel human microarrays with absolute expression values, one-channel human microarrays with differential expression values, and *Streptococcus pneumoniae* two-channel arrays with differential expression values.

We tested four alternative versions of our framework. In each version, either the binary or discrete rank product significance matrix is used; also, we either apply the TF-IDF model before computing cosine similarities between studies, or skip the TF-IDF step and directly compute cosine similarities on the rank product matrix 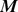. As additional competing methods, we considered correlation-based approaches that, unlike our framework, do not extract any global features from the studies. Instead, these approaches simply estimate the similarity between studies as a combination of the similarity values between microarrays of the compared studies. We chose the Spearman rank correlation coefficient as the similarity measure between microarrays from different studies. We then estimated the similarity between two studies as the maximum, mean, or median of the Spearman correlation between microarrays from the two studies. Our choice of similarity measure is based on the fact that the Spearman correlation coefficient is a well-performing robust nonparametric estimator of statistical dependence [16]. Additionally, it has been previously shown to perform well in the transcriptomics meta-analysis setting [9],[17].

Regarding the actual testing procedure, in essence we measured each method's ability to connect studies that are effectively related, using PubMed MeSH annotations as a gold standard. Given a study designated as the *query*, we sort the remaining studies according to their relevance to the query study. Then, for each of the query study's MeSH terms, we quantify how top retrieved studies tend to include that same MeSH term, using a standard information retrieval performance measure known as Average Precision (AP). Applying the aforementioned test setting yields a set of AP performance scores for every competing method, with every study yielding an AP score for each of its assigned MeSH terms. Throughout this section, we apply a one-sided paired Wilcoxon signed-rank test [16] to evaluate if the AP scores in a given method are significantly higher than the corresponding AP scores in another method. In our context, the one-sided Wilcoxon signed-rank test specifies the null hypothesis that the median difference between paired AP scores from the two methods is zero, while the alternative hypothesis specifies that the median difference is greater than zero.

**Figure 3:**
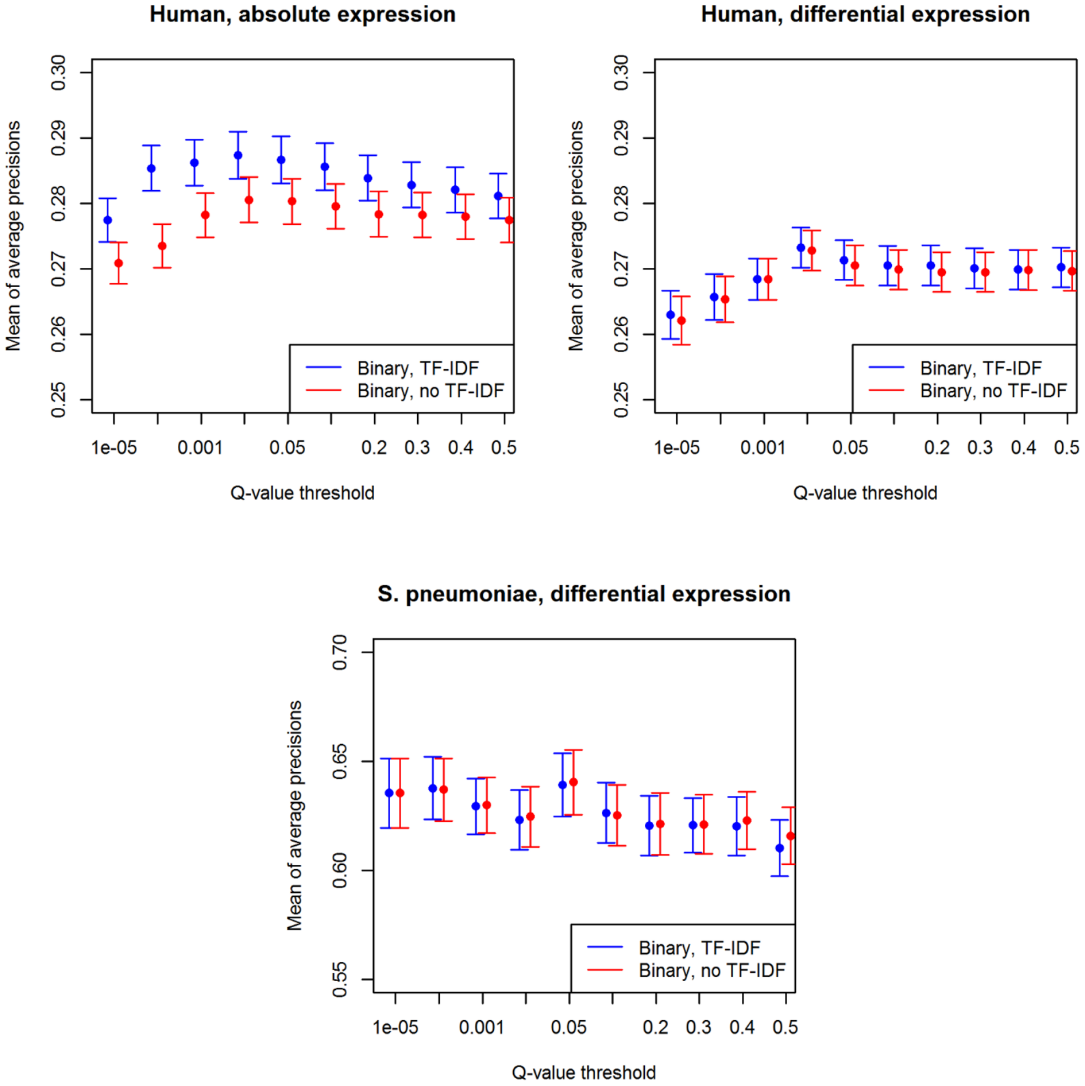
Retrieval performance according to 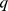-value threshold. Mean and standard error of the AP scores when a 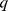-value threshold is used. For each data set, we display the performance for two alternatives of our framework, namely with or without the TF-IDF step. Notice that, for every 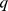-value threshold (x-axis), the two framework alternatives are displayed side-by-side.

The first question we addressed was whether the 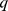-value threshold used in computing the binary significance matrix 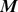 had an impact on the framework's performance. Figure 3 displays the mean and standard error of AP scores when varying the 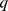-value threshold. It can be seen that the performance peaks at 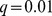 for the human data sets and at 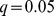 for the *S. pneumoniae* data set, which indicates that standard 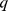-value thresholds may be safely chosen. Additionally, it can be seen that the framework's performance when the TF-IDF step is not performed typically lags behind. The exception is for the *S. pneumoniae* data set, where the “no TF-IDF” framework appears to perform slightly better than the “TF-IDF” framework, particularly at the peak performance point 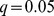. However, the one-sided paired Wilcoxon signed-rank test indicates that this difference is not significant (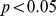). On the contrary, the signed-rank test on the human data set (absolute or differential expression), applied to the data at the peak performance point 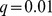, indicates that the “TF-IDF” framework is significantly better than the “no TF-IDF” framework (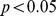). In the following method comparisons, when considering the binary version of our framework, we take 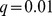 for the human data and 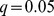 for the *S. pneumoniae* data, in order to avoid displaying an excessive number of performance values for multiple methods.

**Figure 4:**
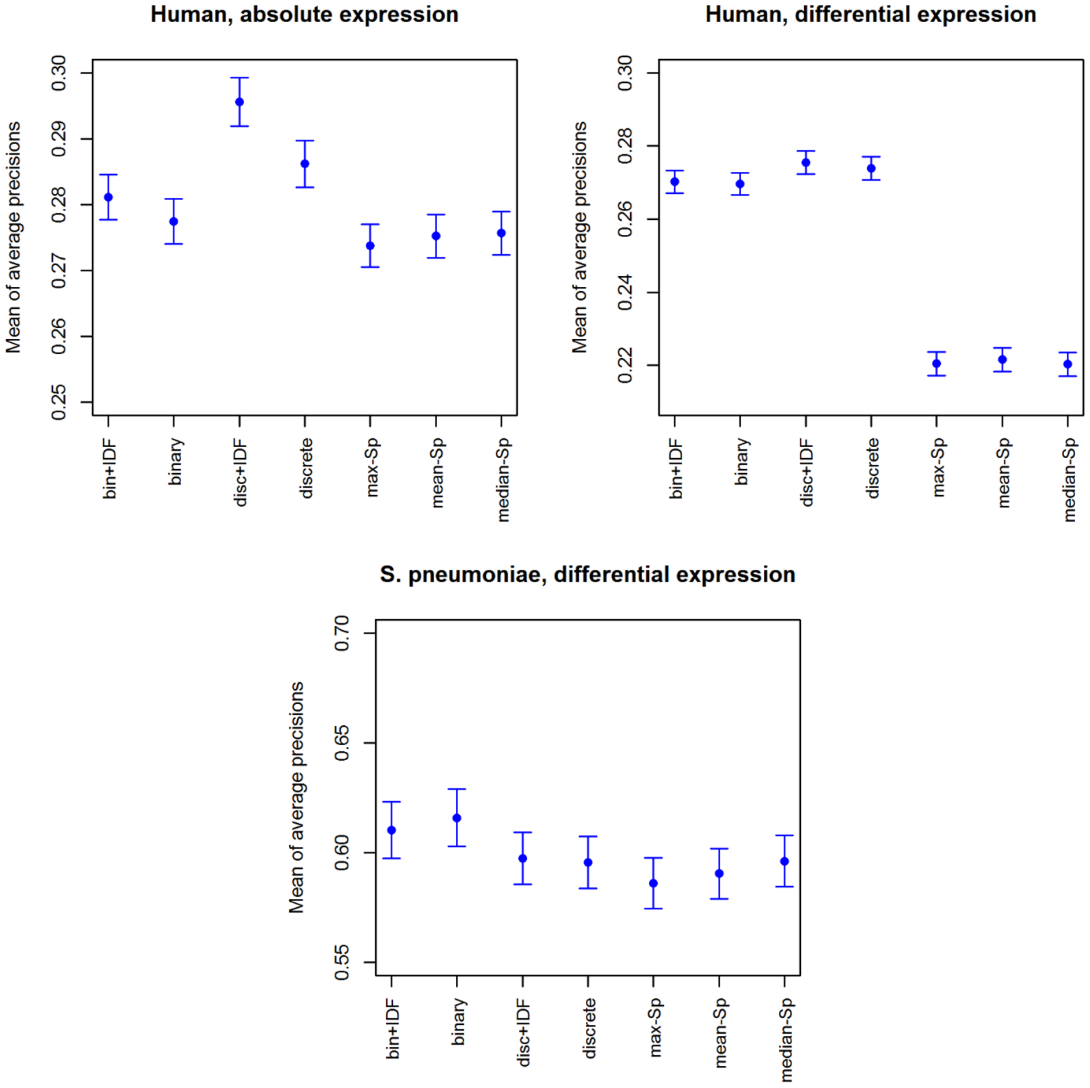
Retrieval performance for all methods. Mean and standard error of the AP scores for four variants of our framework, as well as for three Spearman correlation-based methods. Regarding the x-axis labels, “binary” indicates the binary significance matrix variant of our framework while “discrete” indicates the discrete significance matrix variant; “bin + IDF” and “disc + IDF” indicate those two same variants, but also performing the TF-IDF modeling step prior to establishing connections between studies; finally, the “max-SP”, “mean-SP”, and “median-SP” labels refer to the maximum, mean, and median statistic summarization approaches in the competing Spearman correlation methods.

Then we proceeded to assess the performance differences between our framework variants and the Spearman competing methods. Figure 4 displays the mean and standard error of the AP scores for both our framework and the Spearman correlation-based methods. It can be seen that our framework attains higher scores in all data sets. Finally, is can be seen that on the human data sets, the discrete variants attain a better performance, while in the *S. pneumoniae* data set the binary variant performs the best.

Taken together, these results indicate that our method's feature-based, global encoding is an adequate basis for connecting studies, improving on correlation-based approaches that are known to perform well in the meta-analysis setting [9],[17]. Additionally, usage of the TF-IDF weighting scheme improves upon the connectivity performance of our proposed framework, for both the binary and discrete rank product estimation approaches.

### Biological Interpretation

In this section, we describe case studies using our proposed method with a 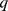-value threshold of 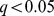.

#### *Homo sapiens* study collection

Table 1 displays query examples, the first two considering absolute expression and the second two considering differential expression. The table also include the corresponding top-three most similar studies and the three protein-coding genes with the highest IDF score that are significant in both the query and the top-three most similar studies. The justification for this heuristic is to focus on genes that are significant in a low number of studies in the collection (*i.e.* that possess a high IDF score); the event of these genes being significant in both the query and the top-three most similar studies is statistically more significant than the same event for genes with a low IDF score. For succinctness of analysis only the three top studies are shown. Note that in order to maximize the interpretability of the results, we display the three *protein-coding* shared genes with the highest IDF scores. In some case studies, the shared probesets with the highest IDF scores do not match to known protein-coding genes. In such cases, we do not present those probesets in the corresponding results table, as interpreting why such probesets are shared by independent studies requires further probeset-mapping work that falls outside the scope of this paper. However, our web-based tool shows the full extent of the results, including probesets that do not match to known protein-coding genes.

**Table 1:** Case studies from the human study collection.

Type	Case study	Results	Details
Absolute expression	Stem cells	Query	microRNA and mRNA expression profiles of human pancreatic islet-like cell clusters (E-GEOD-14503)
		Result #1	Human embryonic stem cells derived from embryos at different stages of development share similar transcription profiles (E-GEOD-29625)
		Result #2	microRNA and mRNA expression profiles of human embryonic stem cells treated with activin A (E-GEOD-16910)
		Result #3	Transcription profiling by array of human embryonic and induced pluripotent stem cells (E-GEOD-23402)
		Top genes	*Cathepsin L2 (CTSL2), von Willebrand factor D and EGF domains (VWDE), ZFP42 zinc finger protein (ZFP42)*
	Muscle tissue	Query	Gene expression profiles in muscle tissue from FSHD patients (E-GEOD-15090)
		Result #1	A transcriptional map of the impact of endurance exercise training on skeletal muscle phenotype (resting muscle after endurance training) (E-GEOD-35659)
		Result #2	Expression data of normal human extraocular muscle and strabismic human extraocular muscle (E-GEOD-38780)
		Result #3	Gene expression in skeletal muscle of cancer patients before and after potentially curative surgery (E-GEOD-34111)
		Top genes	*Mindbomb E3 ubiquitin protein ligase 1 (MIB1), bestrophin 3 (BEST3), myosin light chain kinase family, member 4 (MYLK4)*
Differential expression	Hepatocytes	Query	Transcription Profiling of human primary hepatocytes after treatment with pirinixic acid (E-GEOD-17251)
		Result #1	HepaRG cells as a model of the primary human hepatocyte transcriptome (E-GEOD-18269)
		Result #2	Atorvastatin, rosuvastatin and rifampicin effect on human primary hepatocyte transcriptome (E-GEOD-24187)
		Result #3	Transcriptome Analysis Identifies Fn14, a TNF Superfamily Receptor Member, as a Therapeutic Target in Alcoholic Hepatitis (E-GEOD-28619)
		Top genes	*WD repeat-containing protein 72 (WDR72), indoleamine 2,3-dioxygenase 2 (IDO2), Nuclear Receptor Subfamily 1, Group I, Member 3 (NR1I3)*
	Prostate cancer	Query	Identification of an SRF- and androgen-dependent gene signature in prostate cancer (E-GEOD-22606)
		Result #1	Expression data from androgen treated LNCaP cells (E-GEOD-17044)
		Result #2	Atorvastatin, rosuvastatin and rifampicin effect on human primary hepatocyte transcriptome [Affymetrix platform] (E-GEOD-24187)
		Result #3	Expression data from breast cancer cell lines with various colony-forming ability (E-GEOD-15026)
		Top genes	*Vacuolar protein sorting 13 homolog B (VPS13B), histidine triad nucleotide binding protein 1 (HINT1), diacylglycerol kinase, eta (DGKH)*

ArrayExpress study identifiers are shown next to each study title. The “Type” column indicates if a case study corresponds to absolute or differential expression data. The “Results” column displays for every case study (1) the query study, (2) the top-three most relevant studies, and (3) the three protein-coding genes with the highest IDF score that are active in the query and the top-three most relevant studies.

In the first case study, the query study analyses the expression of pancreatic cell clusters derived from the embryonic stem cell T3 cell line. All of the top three studies are mapped to the MeSH term “Embryonic Stem Cells”. The first shared significant gene is *CTSL2*, a cathepsin family member; it has recently been proposed that Cathepsin L family members play a role in differentiation via histone proteolysis [18]. The second shared gene *VWDE* codes for the *von Willebrand factor* (*VWF*) D and *EGF* domain-containing protein; while no direct role in stem cell differentiation has been proposed for *VWDE*, *EGF* has been shown to promote proliferation of mouse embryonic stem cells in mouse [19], while *VWF* has been shown to regulate adhesion of mesenchymal stem cells to endothelial cells [20]. Finally, *ZFP42* is a known marker for pluripotency in embryonic stem cells [21].

In the second case study, both the query study and the top-three most relevant studies involve expression profiling of muscle tissue. Concerning the three protein-coding genes with the highest IDF score, *BEST3* is expressed in renal and muscle tissues [22]. On the other hand, *MYLK4* is a partly characterized member of the Myosin Light Chain Kinases (MLCKs) protein family, known to have muscle-specific expression [23]. Finally, we did not find evidence for constitutive expression of *MIB1* in muscle tissue, which leaves open the question as to why it is relevant to the association between the three muscle tissue studies. This case study again highlights the ability of the proposed framework to connect independent studies based on tissue-specific expression.

In the third case study, both the query and the retrieved studies concern transcription profiling in hepatocytes. While *NR1I3* is a regulator of multiple hepatic genes [24], we did not find a hepatocyte-related role for *WDR72* or *IDO2*, which suggests these genes may have yet unknown liver-related roles.

In the fourth case study, the framework connects a prostate cancer study with two other cancer studies and one hepatocyte-related study. Concerning the top-three shared genes, *HINT1* is a tumor-suppressor gene in gastric cancer, which suggests it may have a yet-unknown role in prostate cancer [25]. As for *DGKH*, it is known to be associated with bipolar disorder [26], leaving open the question of a potential role in prostate cancer. Finally, we did not find a relevant role for *VPS13B* in the query or retrieved studies. Interestingly, when considering only the query and the top retrieved study, the shared protein-coding gene with the highest IDF score is the transmembrane protease, serine 2 (*TMPRSS2*) gene, known to be differentially expressed in prostate cancer [27].

Finally, we note that when applying the previously described median Spearman correlation method to compute the top-three studies for each query, we were not able to obtain the results here described. Concretely, for the stem cell case study, the retrieved studies were about ulcerative colitis, Parkinson's disease and double-stranded RNA recognition; for the muscle tissue case study, while the top retrieved study is E-GEOD-35659 (*i.e.* the same study as retrieved by our framework), the other two studies are about renal transplantation and pulmonary lymphoma; for the hepatocyte case study, only the study E-GEOD-24187 is retrieved in the top-three studies, the others being about keratinocytes and pancreatic cancer; finally, for the prostate cancer case study, the retrieved studies concern stem cells, interstitial lung disease, and B-cell differentiation, therefore seeming less relevant to the query study than the retrieval results provided by our framework. Additionally, in all case studies the average number of MeSH terms shared between the query and the top-three studies is lower for the median Spearman correlation method. This implies that our framework's improved performance ultimately yields results that are biologically more meaningful than when applying correlation-based approaches.

#### *Streptococcus pneumoniae *study collection

Here, we describe two case studies from the *S. pneumoniae* two-channel microarray study collection related to the MeSH terms “Zinc” and “Virulence”. Table 2 displays each query study, along with the top three most similar studies. In each of these two case studies, we found that the third most similar study does not share the same MeSH term as the query. Therefore, we restricted our analysis to the top-two most similar studies. In general, since the *S. pneumoniae* study collection includes only 21 studies, it is expected that only the very top most similar studies are effectively related to the query.

**Table 2:** Virulence and zinc case studies from the *S. pneumoniae* collection.

Case study	Results	Details
Virulence	Query	Role of PsaR of *Streptococcus pneumoniae* D39 and TIGR4 in global gene expression and virulence (E-GEOD-13505)
	Result #1	Site-specific contributions of glutamine-dependent regulator GlnR and GlnR-regulated genes to virulence of *Streptococcus pneumoniae* (E-GEOD-9850)
	Result #2	CodY of *Streptococcus pneumoniae*: link between nutritional gene regulation and virulence (E-GEOD-7350)
	Result #3	Search for genes essential for pneumococcal transformation: the RADA DNA repair protein plays a role in genomic recombination of donor DNA (E-GEOD-8362)
	Shared genes	mutR, SP_0142, SP_0143
Zinc	Query	Transcriptional response of *Streptococcus pneumoniae* to Zn2+-limitation and the repressor/activator function of AdcR (E-GEOD-29236)
	Result #1	Interplay between manganese and zinc homeostasis in the human pathogen *Streptococcus pneumoniae* (E-GEOD-23504)
	Result #2	The metalloregulatory site in *Streptococcus pneumoniae* AdcR, a zinc-activated MarR-family repressor (E-GEOD-21506)
	Result #3	CelR-mediated activation of the cellobiose-utilization gene cluster in *Streptococcus pneumoniae* (E-GEOD-30891)
	Shared genes	adh, adhP, adcA, adcB, adcC, adcR, lmb, phtD, phtE

Studies are in decreasing order of similarity to the query. ArrayExpress study identifiers are shown next to each study title. The “Results” column displays for every case study (1) the query study, (2) the top-three most relevant studies, and (3) the protein-coding genes that are active in the query and the top-three most relevant studies, in decreasing order of IDF score.

Regarding the zinc case study, both the query and the top-two studies are annotated with the MeSH term “Zinc”. The study samples correspond either to an adhesin competence repressor (adcR) knockout strain vs. wildtype or to a zinc pulse. Zinc is known to influence the expression of multiple *S. pneumoniae* genes [28],[29], while adcR is a zinc-sensing transcription factor involved in multiple processes including virulence and antibiotic stress response [30]. We computed the genes that are significant in all three studies, obtaining a group of nine genes: adh, adhP, adcA/B/C/R, lmb, and phtD/E. Most of these genes have been reported as direct transcriptional targets of adcR in one of the analyzed publications [29]. Our framework therefore confirmed that a group of adcR transcriptional targets is significantly expressed in multiple zinc-related studies. This shows how our meta-analysis approach can be used as an *in silico* computational validation strategy.

Regarding the virulence case study, both the query and the top two studies share the same “Virulence” MeSH annotation. The genes that are significant in all three studies are the transcription factor mutR and two members of a putative bacteriocin system (SP_0142/0143). Interestingly, while large differences in the bacterial virulence phenotype of mouse models were observed in all three studies, no major virulence role has been assigned to any of these genes. It is therefore an open question whether these genes possess a relevant role in the *S. pneumoniae* virulence phenotype. Potential follow-up studies may involve performing transcription and virulence assays of *S. pneumoniae* response to knockouts of mutR, SP_0142 and/or SP_0143.

As in the human case studies, the overlap between our results and the results obtained with the median Spearman correlation method are low — only one study from the virulence case study is also retrieved by the correlation-based method. Additionally, the mean number of MeSH terms shared between the query and the top studies is lower in both case studies for the median Spearman correlation method.

## Conclusions

In the present work we have proposed a framework for performing meta-analysis of transcriptomics studies. Our main contribution is in providing a solution to the open methodological question of whether it is feasible to perform meta-analysis on a global, study-wide level (establishing direct connections between studies rather than between study subsets), by learning study-wide expression signatures that provide a basis for interpreting the connections and deriving novel biological hypotheses.

Using a MeSH-based test setting, we have shown that our framework improves upon the performance of correlation-based measures that estimate the similarity between studies as a combination of the similarities between the corresponding microarrays (this latter approach does not learn any global study features, therefore hindering the subsequent analysis). Finally, via a series of case studies, we showed that our global meta-analysis framework yields biologically meaningful results, in particular suggesting a follow-up study on the potential virulence role of the *Streptococcus pneumoniae* genes mutR, SP_0142, and SP_0143.

Our proposed framework has certain desirable properties: It is fast, since both the rank product and the TF-IDF methods are easily parallelizable and run in a few minutes on a single CPU; it is parameter-free, since apart from an optional, standard 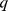-value significance threshold, the user is not required to specify any parameter settings. Importantly, the rank product method is threshold-free, *i.e.*, it does not require the previous specification of a rank threshold that classifies genes in each study sample as “high-ranking” or “low-ranking”. Finally, our log-score interpretation of the rank product method suggests that this method is particularly suited to the high-throughput setting, as log-ranks yield a damping effect that effectively makes large ranks equivalent.

Our proposed test setting was based on the MeSH annotations of the paper associated with each study. However, other ontological tools may be used for evaluation purposes. For instance, the recently developed Experimental Factor Ontology (EFO) [31] maps experimental design variables to standardized terms. The EFO may be used for instance to measure the degree to which similar studies share the same experimental annotations.

One important consideration is how our framework and other recent proposals for gene expression meta-analysis map to “classical” meta-analysis frameworks. In classical meta-analysis, one starts with a set of studies that are known to be related, and methods are proposed for aggregating the results of said studies. Here, the aim is different: There is instead a large, disorganized collection of studies and methods are proposed for suggesting which studies are related. Naturally, the detected connections may correspond to studies that are known to be related and which could be obtained using standard text-driven methods (*e.g.* studies about the same disease). However, the detected connections may also correspond to unexpected, novel connections, which are not supported by the textual annotations assigned to each study, and which may be used as a basis for establishing and testing novel biological hypotheses. Once related studies are identified by our framework, it is straightforward to derive a consensus from the studies, by simply computing the genes which are the most significant in the majority of them.

Finally, the integration of additional data types into our framework is also an open challenge for future work, which is becoming increasingly relevant due the emergence of large-scale transcriptomics projects which aggregate data from complementary technologies [32]. Additionally, statistical meta-analysis methodologies will have to adapt to emerging next-generation sequencing technologies [33]. While our framework takes as input pre-processed gene expression data, therefore not being directly dependent on the underlying technology used to generate the data, applying our framework to next-gen data falls outside the scope of our paper and is presently difficult due to the yet small number of available studies.

## Materials and Methods

### Microarray Data Source

We accessed the ArrayExpress database [2] to download transcriptomics studies from the one-channel Affymetrix GeneChip Human Genome U133 Plus 2.0 and from the species *Streptococcus pneumoniae*. We chose this particular Affymetrix platform because it is the one with the highest number of human studies in ArrayExpress. We also chose *S. pneumoniae* for analysis due to our own expertise and to demonstrate the applicability of our method to two-channel microarrays. For *S. pneumoniae* studies, we focussed our analysis on two-channel microarray studies, which correspond to the vast majority of available studies. We discarded studies that did not contain processed data, which were not associated with published articles indexed in PubMed, or which were a subset of other studies.

For human studies, we restricted our analysis to studies with processed data files under 16 MB, corresponding to about eighty percent of the total number of studies. This threshold was established in order to accelerate the analysis by avoiding the download and parsing of excessively large files.

### Data Processing

We considered microarray studies whose processed expression files contain the standard column names *ID_REF* or *Reporter_Identifier* for identifying probesets and the column names *VALUE* or *AFFYMETRIX_VALUE* for identifying processed expression values. Since *S. pneumoniae* studies correspond to multiple array platforms, we considered only studies whose microarray platform file contains the standard columns *Reporter Name* (probe identifiers) and *ORF* (open reading frame). The column-name requirements allow for a fully automated processing pipeline.

For human studies, we performed the subsequent analysis directly on probesets, while for *S. pneumoniae* studies we first mapped probes to ORFs using each study's corresponding array design file, summarizing the expression of probes that map to the same gene by computing their median expression. We assumed two strategies for missing value replacement, depending on whether the human study collection analysis is based on absolute or differential expression. For absolute expression, we replaced missing values within a given study's expression data with the minimum absolute expression value in that study; for differential expression, we replaced missing values for a given gene with its mean observed expression. The rationale for these two strategies is to assign the lowest ranks to genes whose expression is not observed.

After pre-processing, our approach yields a total of 289 human studies corresponding to 4368 microarray samples and 21 *Streptococcus pneumoniae* studies corresponding to 129 microarray samples.

### *Homo sapiens* Differential Expression

For the human study collection, we performed two independent analyses, one based on absolute expression, the other based on differential expression. In the absolute expression analysis, we did not make use of each study's experimental annotations. In the differential expression analysis, we downloaded each study's experimental design annotation files from ArrayExpress. In those files, each array in each study is assigned to a number of so-called *experimental factor values* which indicate the experimental annotation for that array, *e.g.*, *Disease State = Cardiomyopathy* or *Gender = Male*. We collected all the experimental factor value annotations for all arrays in all studies and manually decided which of those annotations correspond to typical experimental controls (*e.g.*, *DiseaseState = Normal*, *Treatment = Control*, *Time = 0h*). The full list of control annotations is available from Text S1 in File S1. Then, for each study, we selected a subset of arrays corresponding to the study's control. If any arrays in the study are annotated only with experimental control labels, they are selected as the control and averaged. If no such arrays exist, then the control is defined as the average of all arrays in the study. Finally, we compute the log-ratio for each probeset's expression in each of the non-control arrays relative to the corresponding control.

### *Streptococcus pneumoniae *Ortholog Mapping

In order to reconcile *S. pneumoniae* studies from multiple strains, we downloaded reference sequences for strains TIGR4 (NC_003028.3), D39 (NC_008533.1), and R6 (NC_003098.1) from the RefSeq database [34]. We assessed the homology of coding sequences between every pair of strains by running the Nucleotide Basic Local Alignment Tool (BLASTN; [35]). We obtained orthologous gene groups by identifying groups of three genes, one per strain, which are all reciprocal BLASTN best-hits to one another. This approach yields a total of 1621 so-called *S. pneumoniae* metagenes.

### MeSH Terms

We downloaded the entire MeSH tree from the MeSH web site and kept terms from the top-level categories *Anatomy*, *Diseases*, *Chemicals and Drugs*, *Biological Sciences*, and *Persons*. For every study corresponding to a PubMed-indexed paper, we downloaded its associated PubMed MeSH annotations, discarded qualifiers (e.g., *Breast Neoplasms/genetics* and *Breast Neoplasms/metabolism* are treated as equivalent annotations), and kept only the terms that belong to any of the aforementioned MeSH categories. The rationale for only considering a subset of MeSH categories is to discard categories that are not directly about biological findings or the samples and biological/clinical conditions under study (*e.g.* the *Information Science* category, which describes the computational methodology followed in each paper, is not taken into account).

### Rank Product

We obtained study rank data by sorting the genes in each study sample according to their absolute expression (in the case of one-channel absolute expression analysis) or absolute log-ratio (in the case of two-channel arrays or one-channel differential expression analysis). Then we applied the Rank Product method [13] to compute the relevance of each gene in each study. Succinctly, the rank product assigns a score to each gene by computing the product of the gene's ranks across samples in a given study, or equivalently by computing the sum of the log-ranks,
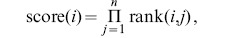
(1)

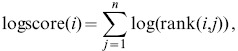
(2)where 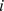 indexes genes, 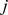 indexes samples in a given study, 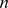 is the total number of samples in a given study, and 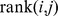 is the rank of the 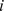-th gene in the 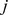-th sample of a given study. The log-function above dampens the distinction between large ranks. We provide an additional discussion of this effect in Text S2 in File S1.

We used a Gamma distribution approximation method to compute 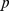-values, which is based on the null hypothesis that ranks are uniformly distributed [36]. Text S2 in File S1 provides the details of the approximate method derivation. More recently, a method has been proposed for computing exact 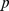-values [37]. Unfortunately, the running time for this method does not scale well enough for our large-scale purposes (Text S2 in File S1).

After obtaining 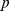-values, we tested two alternatives. First, we computed the corresponding false-discovery rate 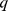-values using the *qvalue* R package [38], and considered genes to be significant at a standard cut-off value of 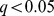. As an alternative, instead of computing a binary significance, we round the value 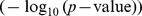 to the nearest integer. This latter alternative yields sparse discrete rather than sparse binary data. Additionally, this latter alternative does not require setting a significance threshold, thus making our framework entirely parameter-free.

The final output of the above approach is either a binary significance matrix 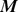 that indicates for every gene-study pair 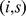 whether gene 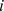 is significant in study 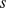,
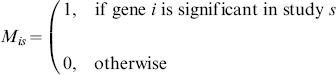
(3)or a discrete matrix 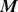 where for each gene-study pair 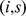, we have 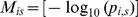, where 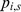 is the 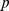-value of gene 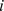 in study 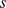. In subsequent sections, we consider a gene 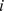 to be *active* in a study 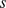 when 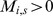.

### Term Frequency–Inverse Document Frequency

In order to detect connections between studies, we model the rank product matrix 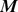 using the Term Frequency-Inverse Document Frequency (TF-IDF) vector space model [39],[40]. The TF-IDF model is commonly used in the natural language processing field; here, each study may be conceptually seen as a “document” and each gene as a “word”.

In the TF-IDF model, each gene receives an Inverse Document Frequency (IDF) score
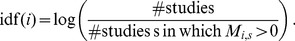
(4)


The IDF score is higher for genes that are active in a lower number of studies, and conversely lower for genes that are active in a greater number of studies. The idea is to model the information content of the event of a gene being active in a given study. Each study 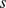 is then encoded as an array 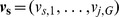, where 
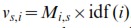
. As a technical detail, if a gene is inactive in all studies, then we simply discard it from the data instead of computing its IDF score. This speeds-up the computations and avoids the problem of having infinite IDF scores.

Finally, the similarity between two studies 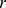 and 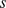 is computed via the standard cosine similarity coefficient which measures the cosine of the angle between the corresponding pair of vectors in the TF-IDF vector space,
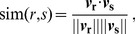
(5)where  is the dot product operator. Genes that possess a lower IDF score contribute less to the dot product between two studies. Conversely, genes that are seldom active in the rank product matrix and which therefore possess a higher IDF score make a larger contribution to the dot product between two studies. In other words, two studies are similar when they both activate genes that possess a high information content.

### Connectivity Performance Evaluation

To assess if the model connects studies that are effectively related, we quantified the relation between study similarity in terms of (5) and study similarity in terms of shared MeSH terms assigned to the studies' PubMed-indexed papers.

For each study (hereafter designated as the query study), we consider each of its assigned MeSH terms in turn. We assume that studies possessing that same MeSH term are relevant to the query study, while the remaining studies are irrelevant. First, we sort all studies according to their similarity to the query study as per (5). Then, we take the similarity-sorted study list and compute the precision 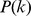 at each rank 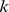 in the list,
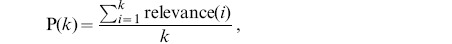
(6)

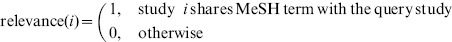
(7)


We then average the precisions at all ranks in the list that correspond to relevant studies,
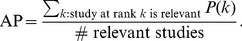
(8)


This measure is known as Average Precision (AP) and is a standard performance measure in Information Retrieval. It can be geometrically interpreted as approximating the area under the precision-recall curve [40].

**Figure 5:**
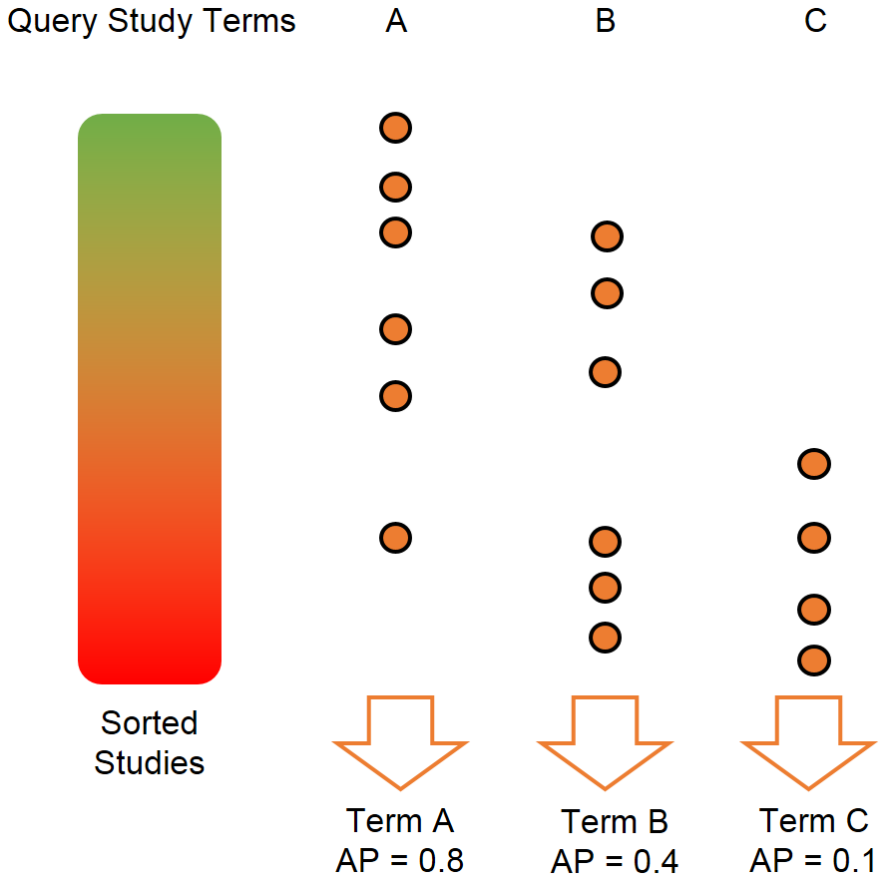
Retrieval performance strategy. Illustration of the proposed validation strategy. Each study in turn is considered as the query study, with the remaining studies being ordered according to their similarity with the query study. The query study has three fictitious MeSH terms: A, B, and C. The brown circles indicate studies that also possess those terms. The Average Precision (AP) performance measure is higher when the most similar studies also possess the given MeSH term, or conversely lower when studies mapped to the MeSH term are among the least similar to the query study. Notice that the number of AP performance scores per query study equals the number of its MeSH terms.

The above procedure yields for every study (considered as the query) and each of its MeSH terms a performance measure between zero and one, that quantifies how the studies that are most similar to the query tend to possess that same MeSH term. Figure 5 illustrates the described performance evaluation strategy.

## Supporting Information

Checklist S1 **PRISMA checklist.**
(PDF)

File S1 **Control annotations and Rank Product technical details.**
(PDF)

## References

[1] Nordmann AJ (2012) Meta-analyses: what they can and cannot do. Swiss Med Wkly 142: w13518. 22407741 10.4414/smw.2012.13518

[2] Parkinson H, Sarkans U, Kolesnikov N, Abeygunawardena N, Burdett T, et al (2009) Arrayexpress update – from an archive of functional genomics experiments to the atlas of gene expression. Nucleic Acids Res 37: D868–D872. 19015125 10.1093/nar/gkn889 PMC2686529

[3] Segal E, Friedman N, Koller D, Regev A (2004) A module map showing conditional activity of expression modules in cancer. Nat Genet 36: 1090–1098. 15448693 10.1038/ng1434 PMC2271138

[4] Sirota M, Dudley JT, Kim J, Chiang AP, Morgan AA, et al (2011) Discovery and preclinical validation of drug indications using compendia of public gene expression data. Sci Transl Med 3: 96ra77. 10.1126/scitranslmed.3001318 PMC3502016 21849665

[5] Huang H, Liu CC, Zhou XJ (2010) Bayesian approach to transforming public gene expression repositories into disease diagnosis databases. Proc Natl Acad Sci USA 107: 6823–6828. 20360561 10.1073/pnas.0912043107 PMC2872390

[6] Lukk M, Kapushesky M, Nikkilä J, Parkinson H, Gonçalves Ângela, et al (2010) A global map of human gene expression. Nat Biotechnol 28: 322–324. 20379172 10.1038/nbt0410-322 PMC2974261

[7] Rhodes DR, Kalyana-Sundaram S, Mahavisno V, Varambally R, Yu J, et al (2007) Oncomine 3.0: Genes, pathways, and networks in a collection of 18,000 cancer gene expression profiles. Neoplasia 9: 166–180. 17356713 10.1593/neo.07112 PMC1813932

[8] Kupershmidt I, Su QJ, Grewal A, Sundaresh S, Halperin I, et al (2010) Ontology-based meta-analysis of global collections of high-throughput public data. PLoS One 5: e13066. 20927376 10.1371/journal.pone.0013066 PMC2947508

[9] Caldas J, Gehlenborg N, Kettunen E, Faisal A, Rönty M, et al (2012) Data-driven information retrieval in heterogeneous collections of transcriptomics data links *SIM2s* to malignant pleural mesothelioma. Bioinformatics 28: 246–253. 22106335 10.1093/bioinformatics/btr634 PMC3259436

[10] Rung J, Brazma A (2012) Reuse of public genome-wide gene expression data. Nat Rev Genet 14: 89–99. 23269463 10.1038/nrg3394

[11] Tseng GC, Ghosh D, Feingold E (2012) Comprehensive literature review and statistical considerations for microarray meta-analysis. Nucleic Acids Res 40: 3785–3799. 22262733 10.1093/nar/gkr1265 PMC3351145

[12] Subramanian A, Tamayo P, Mootha VK, Mukherjee S, Ebert BL, et al (2005) Gene set enrichment analysis: A knowledge-based approach for interpreting genome-wide expression profiles. Proc Natl Acad Sci USA 102: 15545–15550. 16199517 10.1073/pnas.0506580102 PMC1239896

[13] Breitling R, Armengaud P, Amtmann A, Herzyk P (2004) Rank products: a simple, yet powerful, new method to detect differentially regulated genes in replicated microarray experiments. FEBS Lett 573: 83–92. 15327980 10.1016/j.febslet.2004.07.055

[14] Hong F, Breitling R (2008) A comparison of meta-analysis methods for detecting differentially expressed genes in microarray experiments. Bioinformatics 24: 374–382. 18204063 10.1093/bioinformatics/btm620

[15] Lipscomb CE (2000) Medical subject headings (mesh). Bull Med Libr Assoc 88: 265–266. 10928714 PMC35238

[16] Hollander M, Wolfe DA (2000) Nonparametric Statistical Methods. New York, NY: Wiley-Interscience.

[17] Fujibuchi W, Kiseleva L, Taniguchi T, Harada H, Horton P (2007) Cellmontage: Similar expression profile search server. Bioinformatics 23: 3103–3104. 17895274 10.1093/bioinformatics/btm462

[18] Duncan EM, Muratore-Schroeder TL, Cook RG, Garcia BA, Shabanowitz J, et al (2008) Cathepsin l proteolytically processes histone h3 during mouse embryonic stem cell differentiation. Cell 135: 284–294. 18957203 10.1016/j.cell.2008.09.055 PMC2579750

[19] Heo JS, Lee YJ, Han HJ (2006) EGF stimulates proliferation of mouse embryonic stem cells: involvement of ca^2+^ inux and p44/42 MAPKs. Am J Physiol Cell Physiol 290: C123–C133. 16107508 10.1152/ajpcell.00142.2005

[20] Potapova IA, Cohen IS, Doronin SV (2010) Von willebrand factor increases endothelial cell adhesiveness for human mesenchymal stem cells by activating p38 mitogen-activated protein kinase. Stem Cell Res Ther 1: 35. 21083900 10.1186/scrt35 PMC3025437

[21] Shi W, Wang H, Pan G, Geng Y, Guo Y, et al (2006) Regulation of the pluripotency marker *Rex-1* by *Nanog* and *Sox2* . J Biol Chem 281: 23319–23325. 16714766 10.1074/jbc.M601811200

[22] Jiang K, Liu Y, Ma M, Tang YB, Zhou JG, et al (2013) Mitochondria dependent pathway is involved in the protective effect of bestrophin-3 on hydrogen peroxide-induced apoptosis in basilar artery smooth muscle cells. Apoptosis 18: 556–565. 23468120 10.1007/s10495-013-0828-4

[23] Chan JY, Takeda M, Briggs LE, Graham ML, Lu JT, et al (2008) Identification of cardiac-specific myosin light chain kinase. Circ Res 102: 571–580. 18202317 10.1161/CIRCRESAHA.107.161687 PMC2504503

[24] Maglich JM, Lobe DC, Moore JT (2008) The nuclear receptor CAR (NR1I3) regulates serum triglyceride levels under conditions of metabolic stress. J Lipid Res 50: 439–445. 18941143 10.1194/jlr.M800226-JLR200

[25] Huang H, Wei X, Su X, Qiao F, Xu Z, et al (2011) Clinical significance of expression of hint1 and potential epigenetic mechanism in gastric cancer. Int J Oncol 38: 1557–1564. 21468541 10.3892/ijo.2011.994

[26] Baum AE, Cabanero M, Cardona I, Corona W, Klemens B, et al (2008) A genome-wide association study implicates diacylglycerol kinase eta (dgkh) and several other genes in the etiology of bipolar disorder. Mol Psychiatry 13: 197–207. 17486107 10.1038/sj.mp.4002012 PMC2527618

[27] Squire JA (2009) Tmprss2-erg and pten loss in prostate cancer. Nat Genet 41: 509–510. 19399032 10.1038/ng0509-509

[28] Jacobsen FE, Kazmierczak KM, Lisher JP, Winkler ME, Giedroc DP (2011) Interplay between manganese and zinc homeostasis in the human pathogen *Streptococcus pneumoniae* . Metallomics 3: 38–41. 21275153 10.1039/c0mt00050g PMC3061551

[29] Shafeeq S, Kloosterman TG, Kuipers OP (2011) Transcriptional response of *Streptococcus pneu-moniae* to zn(2+) limitation and the repressor/activator function of adcr. Metallomics 3: 609–618. 21603707 10.1039/c1mt00030f

[30] Reyes-Caballero H, Guerra AJ, Jacobsen FE, Kazmierczak KM, Cowart D, et al (2010) The metalloregulatory zinc site in *Streptococcus pneumoniae* adcr, a zinc-activate marr family repressor. J Mol Biol 403: 197–216. 20804771 10.1016/j.jmb.2010.08.030 PMC2949468

[31] Malone J, Holloway E, Adamusiak T, Kapushesky M, Zheng J, et al (2010) Modeling sample variables with an experimental factor ontology. Bioinformatics 26: 1112–1118. 20200009 10.1093/bioinformatics/btq099 PMC2853691

[32] Network CGAR (2008) Comprehensive genomic characterization defines human glioblastoma genes and core pathways. Nature 455: 1061–1068. 18772890 10.1038/nature07385 PMC2671642

[33] Mardis ER (2008) Next-generation dna sequencing methods. Annu Rev Genomics Hum Genet 9: 387–402. 18576944 10.1146/annurev.genom.9.081307.164359

[34] Pruitt KD, Tatusova T, Maglott DR (2007) Ncbi reference sequences (refseq): a curated non-redundant sequence database of genomes, transcripts and proteins. Nucleic Acids Res 35: D61–D65. 17130148 10.1093/nar/gkl842 PMC1716718

[35] Altschul SF, Madden TL, Schäffer AA, Zhang J, Zhang Z, et al (1997) Gapped BLAST and PSI-BLAST: a new generation of protein database search programs. Nucleic Acids Res 25: 3389–3402. 9254694 10.1093/nar/25.17.3389 PMC146917

[36] Koziol JA (2010) Comments on the rank product method for analyzing replicated experiments. FEBS Lett 584: 941–944. 20093118 10.1016/j.febslet.2010.01.031 PMC2849678

[37] Eisinga R, Breitling R, Heskes T (2013) The exact probability distribution of the rank product statistics for replicated experiments. FEBS Lett 587: 677–682. 23395607 10.1016/j.febslet.2013.01.037

[38] Storey JD, Tibshirani R (2003) Statistical significance for genomewide studies. P Natl Acad Sci USA 100: 9440–9445. 10.1073/pnas.1530509100 PMC170937 12883005

[39] Salton G, Wong A, Yang CS (1975) A vector space model for automatic indexing. Commun ACM 18: 613–620.

[40] Manning CD, Raghavan P, Schütze H (2008) Introduction to Information Retrieval. Cambridge, UK: Cambridge University Press.

