# Supplementary material for: Global Meta-Analysis of Transcriptomics Studies
Source: PLoS One. 2014 Feb 26;9(2):e89318. doi: 10.1371/journal.pone.0089318 (PMC3935861; doi:10.1371/journal.pone.0089318)
Supplement: File S1 — Control annotations and Rank Product technical details. (PDF) [file pone.0089318.s002.pdf]

Supporting Information S1
Global meta-analysis of transcriptomics studies

José Caldas[1,*], Susana Vinga[2]
1 INESC-ID, Instituto de Engenharia de Sistemas e Computadores, Investigação e Desenvolvimento, Rua Alves Redol 9, 1000-029 Lisboa, Portugal
2 IDMEC/LAETA, Instituto Superior Técnico, Universidade de Lisboa, Av. Rovisco Pais, 1049-001 Lisboa, Portugal
∗ E-mail: jose@kdbio.inesc-id.pt

Supporting Text S1: Control Annotations

0 hrs

0 minutes

0 weeks

0.0

0h

0hr

0nm

adjacent normal

before methotrexate treatment

control

control 0 min

control cells

control plasmid

control sirna

dmso control

dmso vehicle

empty vector (pcdna3.1)

euploid

healthy

healthy control

healthy donor

il-4 treatment for 0h

mismatch mm control

mock

mock infected

naive

negative control

no cytokine

non-rejecting

non-smoker

non-sp

non-targeting control sirna

non tol

none

normal

normal liver obtained from a healthy adult subject who suffered sudden death

p53 wild type

p53 wildtype

placebo

shcontrol

skin non lesion

surrounding noncancerous liver tissue

undifferentiated

unsorted

untreated

untreated control

vector

vehicle

vehicle control

week 0

wild-type

wild-type u2af35

wild type

wildtype

wildtype bt474 cells

wt

Supporting Text S2: Rank Product

Significance Estimation

As mentioned in the main text, we used a Gamma distribution approximation method to compute the rank product p-values, which is based on the null hypothesis that ranks are uniformly distributed [1]. Here, we describe the approach used by [1].

Succintly, if a given gene rank divided by the number of genes is approximately uniformly distributed (the rank is discrete, not continuous, so this is necessarily an approximation), then its negative logarithm is exponentially distributed,

$$\frac{r_{ij}}{G+1} \approx \text{Unif}(0,1)\,, \tag{1}$$

$$-\log \frac{r_{ij}}{G+1} \approx \text{Exp}(1)\,, \tag{2}$$

where G is the total number of genes. In the equation above, the normalizing factor $G+1$ instead of G was used, so that the expectation of the uniformly distributed normalized rank is $K/2$ instead of $(K+1)/2$.

The sum of log-ranks that constitutes the rank product score for a given gene i is therefore related to the sum of exponentially distributed variables,

$$\text{logscore}(i) = \sum_{j=1}^{n} \log r_{ij} \tag{3}$$

$$= n\log(G+1) + \sum_{j=1}^{n} \log \frac{r_{ij}}{G+1}. \tag{4}$$

The sum of n exponentially distributed variables with unit expectation is equal to a Gamma-distributed variable with shape n and unit scale. Therefore, in order to compute the probability that a given log-rank sum is smaller than an observed value (we remind the reader that in the present context, smaller ranks are better), one needs to compute the lower tail of the corresponding Gamma distribution,

$$P(\text{logscore}(i) < x) = P\left(n\log(G+1) + \sum_{j=1}^{n} \log \frac{r_{ij}}{G+1} < x\right) \tag{5}$$

$$= P\left(\sum_{j=1}^{n} \log \frac{r_{ij}}{G+1} < x - n\log(G+1)\right) \tag{6}$$

$$= P\left(-\sum_{j=1}^{n} \log \frac{r_{ij}}{G+1} > -x + n\log(G+1)\right) \tag{7}$$

$$= P\left(\text{Gamma}(\text{shape} = \text{n}, \text{scale} = 1) > -x + n\log(G+1)\right)\,. \tag{8}$$

In summary, to compute the p-value for a given gene log-score x, we compute the term $-x+n\log(G+1)$ and then compute the probability that a Gamma-distributed variable with scale n and unit shape exceeds the computed term.

Log-Score Interpretation

One relevant aspect of the rank product method is that the log-score assigned to each gene and used for determining significance consists of a sum of log-ranks,

$$\text{logscore}(i) = \sum_{j=1}^{n} \log(\text{rank}(i,j)). \tag{9}$$

For a large rank r, $\log(r) \approx \log(r+1)$, implying that the terms in (9) corresponding to large ranks are mostly indistinguishable from one another. For instance, for ranks $r_1 = 1000$ and $r_2 = 5000$, we have that $\log r_1 \approx 6.90$ and $\log r_2 \approx 8.52$, *i.e.*, while r_2 is five times larger than r_1, its logarithm in only about 23% larger than the logarithm of r_1. To the best of our knowledge, this interpretation of the rank product method has not been provided before.

Intuitively, the above discussion means that the terms in the log-score are softly divided into terms corresponding to lower ranks and terms corresponding to larger ranks (which possess similar log-values). The idea of providing a soft distinction between low-ranks and the rest has been previously used in statistical testing frameworks for gene expression [2].

Computing Exact p-values

A method for computing exact p-values has been recently proposed [3]. Here, we measure the method's running time requirements on Windows 7 R, using an Intel i7-3610QM CPU at 2.3Ghz.

We assume that a given study has n genes and k samples. For the human differential expression study collection, the median number of genes and samples is $n = 9701$ and $k = 12$. For the *S. pneumoniae* study collection, the median number of genes and samples is $n = 1935$ and $k = 6$. We perform our benchmarking using these values.

The exact method relies on explicitly computing the number of possible ways in which the product of a gene's ranks across a study's samples is exactly equal to a given rank product value x. This number is designated as $H(x; k, n)$. The exact unnormalized p-value for a given rank product x can be obtained by computing the total number of ways in which the rank product is less than or equal to x, *i.e.*, $H(1; k, n) + H(2; k, n) + \ldots + H(x; k.n)$. The normalized p-value is then obtained by dividing by n^k, which is the total number of possible rank combinations of a gene across the k samples. The p-value computation pseudo-code is thus as follows:

1. $p \leftarrow 0$
2. For $i = 1, \ldots, x$:
   (a) $p \leftarrow p + H(i; k, n)$
3. $p \leftarrow \frac{p}{n^k}$

We chose as a benchmark task the time it takes for the code above to reach a p-value which is deemed significant using Bonferroni correction at an initial p-value of 0.05. For the human study collection, this p-value threshold is $p = \frac{0.05}{9701}$, while for the *S. pneumoniae* study collection it is $p = \frac{0.05}{1935}$. Intuitively, we recreate the time it takes for the exact method to return the p-value for a gene whose rank product score is barely significant using Bonferroni correction. For both the human and the *S. pneumoniae* study collection, the exact method takes over forty minutes to compute. Given the impractical running time for the exact p-value method, we have opted for using the Gamma approximation method, which runs instantly. However, in the future it may be possible to combine both approaches in order to obtain a fast and precise hybrid method.

References

1. Koziol JA (2010) Comments on the rank product method for analyzing replicated experiments. FEBS Lett 584: 941–944.

2. Subramanian A, Tamayo P, Mootha VK, Mukherjee S, Ebert BL, et al. (2005) Gene set enrichment analysis: A knowledge-based approach for interpreting genome-wide expression profiles. Proc Natl Acad Sci USA 102: 15545–15550.

3. Eisinga R, Breitling R, Heskes T (2013) The exact probability distribution of the rank product statistics for replicated experiments. FEBS Lett 587: 677–682.
